# Nævi

**Published:** 1895-07-06

**Authors:** W. McAdam Eccles

**Affiliations:** Assistant Surgeon West London Hospital and City of London Truss Society, Surgeon St. Marylebone General Dispensary, Senior Assistant Demonstrator of Anatomy St. Bartholomew's Hospital.


### MEDICAL PROGRESS AND HOSPITAL CLINICS.

[*The Editor will be glad to receive offers of co-operation and contributions from members of the profession. All letters should be addressed to* THE EDITOR, THE LODGE, PORCHESTER SQUARE, LONDON, W.]

### NÆVI.

By W. McADAM ECCLES, M.B., M.S. Lond., F.R.C.S. Eng., Assistant Surgeon West London Hospital and City of London Truss Society, Surgeon St. Marylebone General Dispensary, Senior Assistant Demonstrator of Anatomy St. Bartholomew's Hospital.

So common are nævi that the very word itself, although purely technical, has passed into common everyday language, and this, together with the unsightliness of the affection, is ample apology for its being made the subject of a short article.

A nævus (Latin for a mole) may be situate in almost any region of the body, and more than one may be present in the same subject. It has been a matter of discussion as to whether they are invariably congenital, but from the bulk of the evidence adduced it would seem that they seldom if ever arise after birth, though in early life they may be so minute as to escape observation. They undoubtedly have a marked tendency to increase in size after birth, and may in some rare cases assume enormous proportions. It is a matter of mere accident if they be associated with other deformities or defects.

There are two well-marked varieties of nævi, which must be very familiar to all practitioners—the capillary nævus or plexiform angioma, and the venous nævus or cavernous angioma; but it must be clearly understood that these two forms are very frequently associated with one another. In the lay mind there resides an impression that nævi are highly dangerous because of a reputed tendency to hæmorrhage. Undoubtedly there must have been some cases where serious bleeding has occurred, and as is usual, the exception has taken the foremost place; whereas, on the other hand, thousands of instances could be found in which no untoward symptoms have arisen.

The capillary or cutaneous nævus if left to itself will probably in the end become spontaneously cured, islets of white, more or less healthy, skin appearing in the patch, and gradually spreading or coalescing until the whole area resumes its normal condition.

The subcutaneous or venous nævus is less likely to undergo shrinkage, and commonly steadily progresses.

Capillary nævi are always found in the skin or mucous membranes. The cause of their origin and growth is quite uncertain. They consist essentially of capillary vessels greatly increased in number and size, and at the same time very tortuous and held together by a scanty amount of connective tissue. They appear as slightly elevated vascular growths, having a bright red or purplish tint, and becoming very much paler on pressure being applied, but resuming their colour on its removal.

The variety known as "port wine mark" is peculiarly prone to attack the face, and there to cover not inextensive areas, causing much disfigurement. In the treatment of superficial capillary nævi the question first to be answered is whether active measures shall be taken or whether the case shall be left alone. The position, the size, and the rate of growth are all factors which should be weighed in determining the line of practice to be followed. It is well to bear in mind also that the patient's friends often look upon the disease in a serious light, and are very desirous of its cure. They should be reassured that it is but very rarely that any untoward result arises as the outcome of a capillary nævus, and that small ones which exhibit little or no tendency to increase in size, and are not on a part of the body where they cause disfigurement, may be left alone, with the probability that they will spontaneously disappear. On the other hand, if the nævus is of wider extent, tends to grow, and is obviously unsightly, an attempt should be made to cure it. Port wine marks hardly ever admit of any very satisfactory treatment, though scarifying the region may bring about some cicatrisation and so obliteration of the cutaneous vessels, and in some cases electrolysis may benefit. An ordinary capillary nævus may be readily destroyed by ethylate of sodium or nitric acid, if small by one application, if larger by treating only a portion at a time. In the use of these caustics it is well to take care that only the affected area be treated, the skin around being protected by the application of vaseline. The actual cautery is also useful in many cases.

The objections to these methods of treatment are that they are painful and tend to leave an ulcerating surface after the destruction of the actual nævoid tissue. Lately I have been employing, with markedly gratifying results, a saturated solution (40 per cent.) of formic aldehyde in water. This has the property of tanning animal tissues, is an antiseptic, and causes no pain. It requires, however, very repeated application to render it effective. I generally order that the nævus be painted with it at least thrice daily. It is especially useful for nævi in certain regions, such as over a fontanelle and about the genital organs. I have found after a week or so steady treatment the nævus has become a small mass of hardened tissue. Let me say that this reagent is only of use in quite superficial nævi of no large extent.

The venous nævus when subcutaneous forms most usually a definite tumour and demands prompt treatment, the three chief methods employed are—(1) electrolysis, (2) ligature, (3) excision.

1. *Electrolysis.*—This is undoubtedly one of the very best modes of treatment, its chief advantages being it leaves but little scarring, if properly employed, and does not cause hæmorrhage or much after-pain. The drawbacks to its use are the special apparatus requisite, the length of time it takes to effect a complete destruction of the nævus, and the considerable experience needed in order to secure an entirely satisfactory result from its application. The appliances needed are a battery of some twenty cells of the Leclanché type, a galvanometer reading up to 150 milliampères, insulated connecting wires, and numerous platinum needles, or one or other of the specially devised multiple needles in use. In performing the operation the patient, especially if a child, should be under the influence of chloroform. In an adult cocaine may be used if the area under the electrolytic action be small. There are some highly important details to remember in the process of cure. The parts should be rendered aseptic, and the needles introduced well into the nævoid tissue before any current is passing. The proper insertion of the needles is desirable. They should be parallel to one another, and on no account allowed to touch when inserted, or to come in contact after introduction. They should pass in the whole length of the uninsulated portion, and thus it is necessary to have needles of different lengths. No part of the exposed needle should be lying upon the sound skin. The current is then very gradually and steadily raised from zero up to 40, 60, or even 100 milliampères, all the while its effect on the nævus is closely watched. Around the needles a marked change in colour will be noticed, the positive being the seat of coagulation of the blood and pallor, while the negative have gas evolved from them. As soon as the tissue tends to become blackened, which first occurs at the negative pole, it is time to alter the position of the needles, and it is well to gradually diminish the current to zero before doing so. Any sudden change in the intensity, or better density, of the current, or any contact of the needles will cause a shock to the patient which is by no means without danger in young children under an anæsthetic. If the nævus be very extensive, it must be dealt with in parts, and generally at different sittings. In withdrawing the needles no current should be flowing. The positive needles may cause a little trouble in their dislodgement, while the negative are usually quite loose. A very little hæmorrhage occasionally occurs. The after treatment is simple, a pad of aseptic wool being applied, and if by any chance suppuration should occur boracic fermentations are indicated. If the treatment has been efficiently and satisfactorily carried out there will be very little scarring, even if the nævus be partly cutaneous. It is, however, well to warn the patient's friends that some disfigurement must necessarily remain, and that the treatment may unavoidably lead to some cicatrisation. Electrolysis has been very successfully applied to nævi which occur in situations where any other treatment is practically out of the question.

2. *Ligature.*—It is certain that this method of treatment, which was at one time very much in vogue, has given place considerably to electrolysis. It is, however, a very effectual means of cure. The ligature requires to be applied in various ways, according to the size and position of the nævus. Boiled (aseptic) silk of the Chinese twist variety is the best material to use. In these operations, again, the parts dealt with must be carefully cleaned and sterilised with an antiseptic. In applying the ligature it is to be passed beneath the growth with an ordinary suture needle, or a special nævus needle mounted on a handle. The sound skin should be watched so that it is not included in the noose. One objection to this method is the after pain caused by it.

3. *Excision.*—For small nævi this is an ideal treatment, and more especially in those forms which are associated with lipomatous tissue. It has always to be remembered that this method may give rise to rather serious and troublesome hæmorrhage, unless the limits of the nævoid tissues be well avoided. Nævi of the lip may be very conveniently removed by the knife.

